# 

**Published:** 1898-07

**Authors:** 


### MARRIAGES.

At Kansas City, Mo., June 22, 1898, Dr. Thomas Jackson Turner to Miss Ella Bates. Dr. Turner is a graduate of the American Veterinary College, ex-State Veterinarian of Missouri, ex-Secretary of the United States Veterinary Medical Association, an attache of the Bureau of Animal Industry, now located at Indianapolis, Ind., where the newly married couple will make their home after July 15, 1898.

### PERSONALS.

Among the veterinarians at the recent stallion show, West Chester, Pa., were to be noted: Drs. James B. Rayner, Charles F. Oat, West Chester; Harry P. Turner, Lahaska; J. C. Bartholomew, Berwyn; W. L. Rhoads, Lansdowne; and W. P. Phipps, Lionville.

Veterinarian S. G. Hendren, formerly of York, Pa., is now located at Indianapolis, Ind.

Dr. E. S. Muir, of Philadelphia, Pa., is a devotee of the wheel, and wears the laurels of successful century runs.

Dr. J. C. McNeil, of Pittsburg, Pa., is a member of the city school-board and president of the board of directors of his district, which is said to be the most progressive and advanced of the Smoky City.

Dr. Farrell, of Chicago, has been inspecting horses for the army during the latter part of May and June. It is said that as many as fifty-two head of horses were passed in fifty-nine minutes.

Dr. J. J. Vandervee, of Cornell, Ill., fills the rôle of police magistrate, notary public, and real-estate agent in his bailiwick.

Dr. W. C. McMackin, of Raleigh, N. C., contributed to the Department at Washington three balls of stiff hairs of mature crimson clover taken from the intestines of horses.

Veterinarian G. Howard Davidson, of First Regiment, Squadron A, of New York State, was a recent visitor to Kentucky for the purchase of horses suitable for army service of his troop.

Drs. Harry Walter, of Wilkesbarre, and J. C. McNeil, of Pittsburg, Pa., were visitors to Philadelphia in June.

Dr. Leonard Pearson, State Veterinarian of Pennsylvania, was a visitor to Boston in June.

Dr. T. M. Owen, graduate of the Ontario Veterinary College, who has recently been practising in the Nutmeg State, has located at Gaithersburg, Md.

Drs. R. A. Dunn, of Titusville, and J. T. Ferley, graduates of the class of 1892 of the American Veterinary College, were visitors to the JOURNAL office in June while renewing their fraternal relations.

Dr. Donald McIntosh, of Urbana, is a contributor to the annual publication of the Agricultural Club of the University of Illinois, entitled the *Illinois Agriculturist.*

Dr. A. S. Alexander, of Evanston, has an article in the June 1st number of the *Breeders' Gazette* on the so-called "Cornstalk Disease."

Dr. E. H. Brown, graduate of McKillip Veterinary College, is located at Boise, Idaho, a State that can boast of but few qualified members of the profession.

Dr. W. G. Hollingsworth, of Utica, N. Y., reports a second case of purpura hemorrhagica recovering under the antistreptococcus-serum treatment.

Dr. M. O'Connell, of Holyoke, Mass., has been seriously ill for the past three months, but we are glad to report his convalescence.

Dr. A. B. Kelly, graduate of the class of 1898, New York State Veterinary College, has located at Cohoes, N. Y.

Dr. E. Schirmer, of Washington, D. C., has gone to Tampa, Fla.

Drs. E. M. Massinger and Lewis Ralston, of Phœnixville, Pa., were visitors to the JOURNAL office in June.

Dr. A. T. Peters, of Lincoln, is a contributor to the annual report of the Nebraska Dairymen's Association.

Dr. A. W. Clement, of Baltimore, Md., was a visitor to Philadelphia in June.

Dr. J. P. Turner, late of Fort Myer, Va., was a visitor to West Chester, Philadelphia, and other Eastern cities in June.

Dr. W. P. Phipps, of Lionville, Pa., is the owner of the trotting stallion " Lord Middleton, by " Star Wilkes," and exhibited him at the recent stallion-show in West Chester.

Dr. A. N. Lushington, of Philadelphia, Pa., graduate of the University of Pennsylvania, Veterinary Department, left in June to assume the duties of instructor in the elementary principles of veterinary and sanitary sciences and hygiene at the Belmear Industrial School near Rock Castle, Va.

Dr. W. H. Bolyn, of Lincoln, Va., has been selected as veterinarian for the Upperville Colt-club of that State.

Dr. J. C. Bartholomew, of Berwyn, Pa., is an ardent admirer of the hackney. At the West Chester stallion show he exhibited " Norway," by " Cadet," and " Campfire," by " Wildfire," and " Llemer," a pure Arab stallion.

Dr. J. O. George, of Camden, N. J., has just been re-elected meat-inspector of that city.

Dr. J. J. Monyhan, of Holyoke, Mass., was unfortunate enough in June to lose a valuable pocket-case of instruments.

Dr. C. H. Magill, of Philadelphia, was the consignor of a " Director " colt at McFarland's special sale of trotters and high-class horses in June.

Dr. Leonard Pearson spent the 4th of July at the farm of Dean Marshall, of the Medical Department of the University, in Berks County.

Dr. James M. Mecray, of Maple Shade, N. J., is much interested in the rearing of guinea-pigs, and has specimens of the several varieties in his pens.

Dr. Leonard Pearson, of Philadelphia, has been appointed by Governor Hastings a delegate to the Congress on Tuberculosis convening in Paris July 27 to August 2, 1898.

Oliver Warren, veterinary surgeon, of Victor, N. Y., died the latter part of June. He was a successful gelder in his day, and a great admirer, owner, trainer, and driver of trotting-horses.

Dr. W. B. Niles, of Ames, Ia., presented a paper on "Some of the Diseases of Pigs" before the Iowa Swine-breeders' Association in the latter part of June.

Dr. S. G. Hendren, formerly of York, Pa., recently of Indianapolis, Ind., and Dr. Thos. Castor, of Frankford, Philadelphia, have been granted furloughs by the Department of Agriculture and have entered the engineer corps of the regular army for service in the Hispano-American war.

Dr. Frank Winant, of Ellenville, N. Y., has entered the government service and gone forth prepared to battle for our nation.

Dr. J. W. Petty, of Winston, N. C., was on the sick-list for several weeks in April.

Dr. R. S. Huidekoper, of New York, was a visitor to Philadelphia in May.

Dr. Robert Ward, of Baltimore, has been appointed a member of the Maryland State Board of Examining Horseshoers.

The Governor of Maryland has made the following appointments upon the State Board of Veterinary Medical Examiners: Drs. A. W. Clement, W. H. Martenet, and H. A. Meisner, of Baltimore, F. H. Mackie, of Fair Hill, and R. V. Smith, Frederick City.

Drs. Albert Drinkwater, Leroy R. Webber, and J. C. McKenzie lectured to the horseshoers of Rochester the past winter.

Dr. Leonard Pearson addressed the Farmers' Institute at Huntingdon, Pa., and the annual meeting of the Pennsylvania Jersey Cattle-breeders' Association in March.

Dr. W. E. Weihe, of Scranton, Pa., made a Southern trip in the latter part of February.

Dr. W. H. Dalrymple, of Baton Rouge, has much of interest in a recent bulletin of the State work.

Dr. M. A. Piche, of Montreal, renders a strong protest against the present control and direction of the veterinary sanitary police measures of the Province of Quebec.

Dr. A. Smith, of Toronto, has been elected Vice-President of the Horse-breeders' Association of Canada.

